# Bloody Pericardial Effusion with a Huge Pericardial Mass: A Case Report

**Published:** 2020-01

**Authors:** Arezou Zoroufian, Mahmoud Shirzad, Narges Shahbazi, Mohammad Saheb Jam, Zahra Rahnamoun, Shapoor Shirani, Tahereh Davarpasand

**Affiliations:** *Tehran Heart Center, Tehran University of Medical Sciences, Tehran, Iran.*

**Keywords:** *Heart neoplasms*, *Thymoma*, *Echocardiography*, *Pericardial effusion*

## Abstract

Nowadays, the early diagnosis of tumoral diseases is more possible and accurate with multiple diagnostic imaging modalities such as chest X-ray, echocardiography, computed tomography, and magnetic resonance imaging, especially for cardiac tumors which are usually asymptomatic, even in large sizes. In cardiac masses, the patients’ presentations are non- specific and dependent on the tumor size and site as well as its compressive effect on the adjacent structures. On the other hand, the first and last signs could be sudden cardiac death. However, cardiac masses are either benign or malignant and metastatic in their malignant type, and their definite diagnosis is only possible by surgical tumor resection and tissue biopsy. In this paper, we describe an old patient with severe pericardial effusion and an unusual intrapericardial tumor in transthoracic echocardiography, representing a rare case of a giant ectopic thymoma after surgical resection and pathologic assessment.

## Introduction

Thymomas are rare anterior mediastinal tumors that arise from the thymic tissue. They can be asymptomatic and benign or malignant with fatal invasion to the adjacent mediastinal structures. Ectopic thymomas originate from migrated embryonic thymic cells and can be found in the neck, lungs, and pleural and pericardial spaces. With regard to their unusual site, the most important point is to differentiate them from other mediastinal tumors.^1^^-^^3^ Different diagnostic imaging modalities such as chest X-ray, echocardiography, computed tomography (CT), and magnetic resonance imaging can be helpful in differential diagnostic and prognostic purposes.^4^ We herein present a very rare case of a giant ectopic thymoma, as an unusual intrapericardial tumor, in an old man with a complaint of dyspnea and cardiomegaly in his chest X-ray.

## Case Report

**Figure 1:**
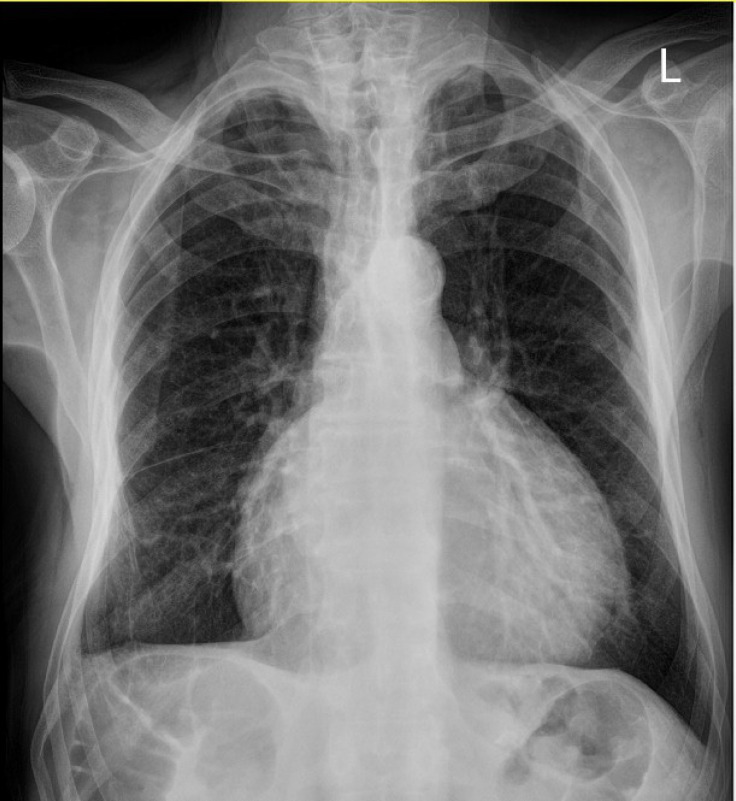
Cardiomegaly in chest X-ray (posterior-anterior view)

A 73-year-old man with a history of controlled hypertension and progressive dyspnea of 3 months’ duration (New York Heart Association functional class II) and recent nonproductive coughs referred with the water-bottle heart sign in his chest X-ray, suggesting pericardial effusion (Figure 1). Physical examinations revealed normal vital signs and oxygen saturation of 94% without an oxygen supply. The remaining physical examinations were unremarkable. The patient did not complain of systemic symptoms such as weight loss, fever, anorexia, and fatigue. Electrocardiography showed atrial fibrillation rhythms and nonspecific ST-T changes. Laboratory tests including complete blood count, renal and hepatic function tests, urine analysis, the coagulation profile, and inflammatory markers (the erythrocyte sedimentation rate and C-reactive protein) were all within the normal ranges. 

**Figure 2:**
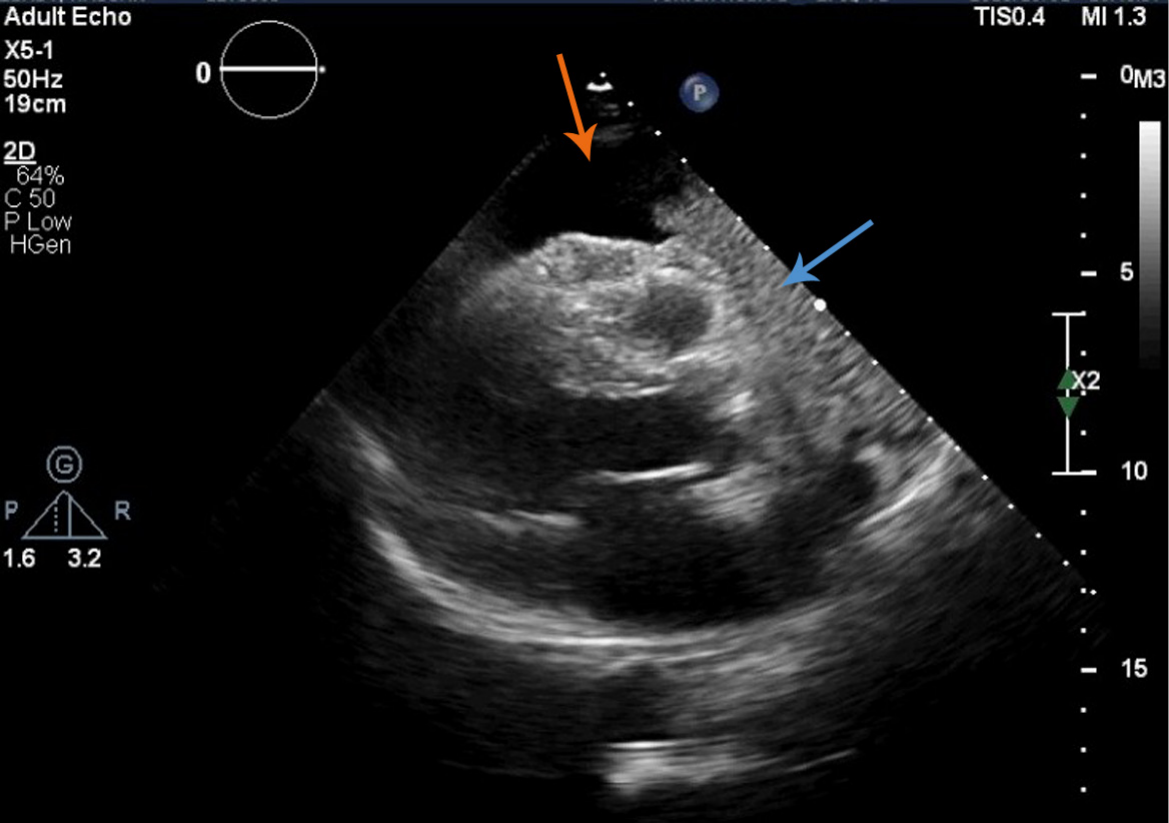
Severe pericardial effusion (orange arrow) and a large mass (blue arrow) anterior to the right ventricular outflow tract in the parasternal long-axis view in 2D transthoracic echocardiography

A 2D transthoracic echocardiographic examination showed severe circumferential pericardial effusion up to 25 mm anterior to the right ventricular outflow tract without right atrial and right ventricular collapse (Figure 2 & Video 1). Additionally, a large non-homogenous intrapericardial mass was visible on the anterior side of the right ventricular outflow tract without obvious compressive effects on the cardiac chambers. The other echocardiographic parameters were within the normal range except moderate mitral and tricuspid regurgitation.

**Figure 3:**
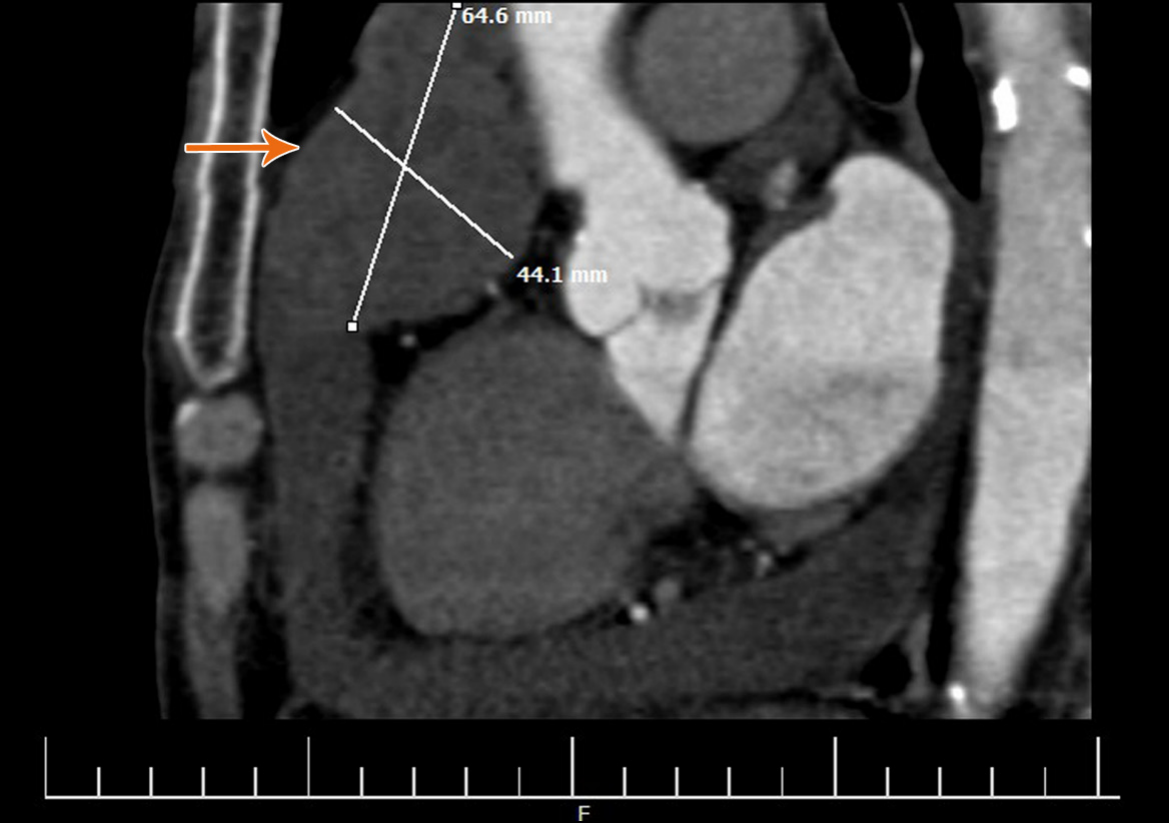
Coronal view of the mediastinal mass (orange arrow) in multislice spiral thoracic computed tomography scan with contrast

To determine the extension and the invasion of the mass, we performed a multislice spiral thoracic CT scan with contrast and found a large ill-defined non-enhancing mass. The mass was an intrapericardial lesion adjacent to the right atrium and had an extension toward the right ventricular outflow tract and the base of the ascending aorta (Figure 3). Except for severe pericardial effusion, no other lesions were noted in the chest or the upper abdomen. Finally, with the suspicion of a soft tissue tumor, the patient was referred for thoracotomy and mass resection after consultations with a cardiac surgeon.

**Figure 4:**
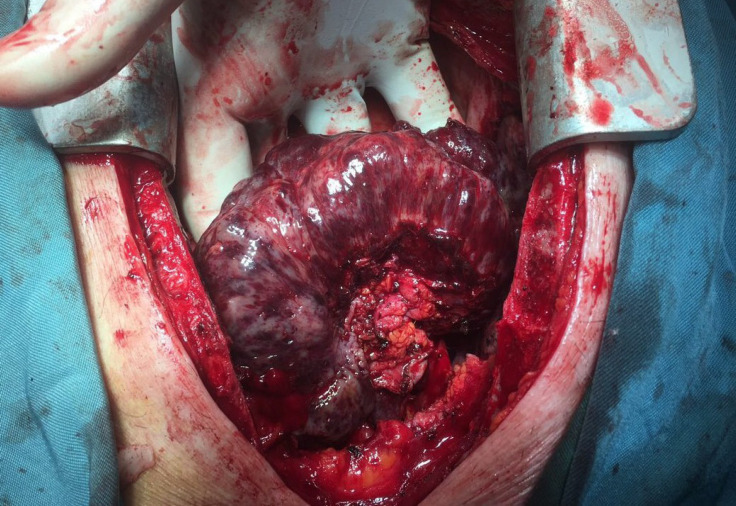
Gross appearance of the pericardial mass in the operating room after total resection

Following general anesthesia and mid sternal thoracotomy, pericardiocentesis was done for a 500-cc bloody pericardial fluid. A large mass, 13 cm in the greatest dimension, was discovered (Figure 4). The mass was attached to the pericardium over the ascending aorta and the right atrium. On gross examination, the tumor was partially encapsulated. In some areas, the capsule was invaded (stage IIb according to the Masaoka staging system). The tumor was brownish in color with the spongy solid consistency of cut surfaces. The areas of hemorrhage and necrosis were visualized. After total mass resection and pericardial biopsy, the specimen was sent to the surgical pathology department for histopathology and immunohistochemical staining in a 10% buffered neutral formalin solution.

The surgery was uncomplicated, and the patient was discharged in good general condition. His next visit was scheduled for 4 weeks later in the outpatient clinic.

**Figure 5:**
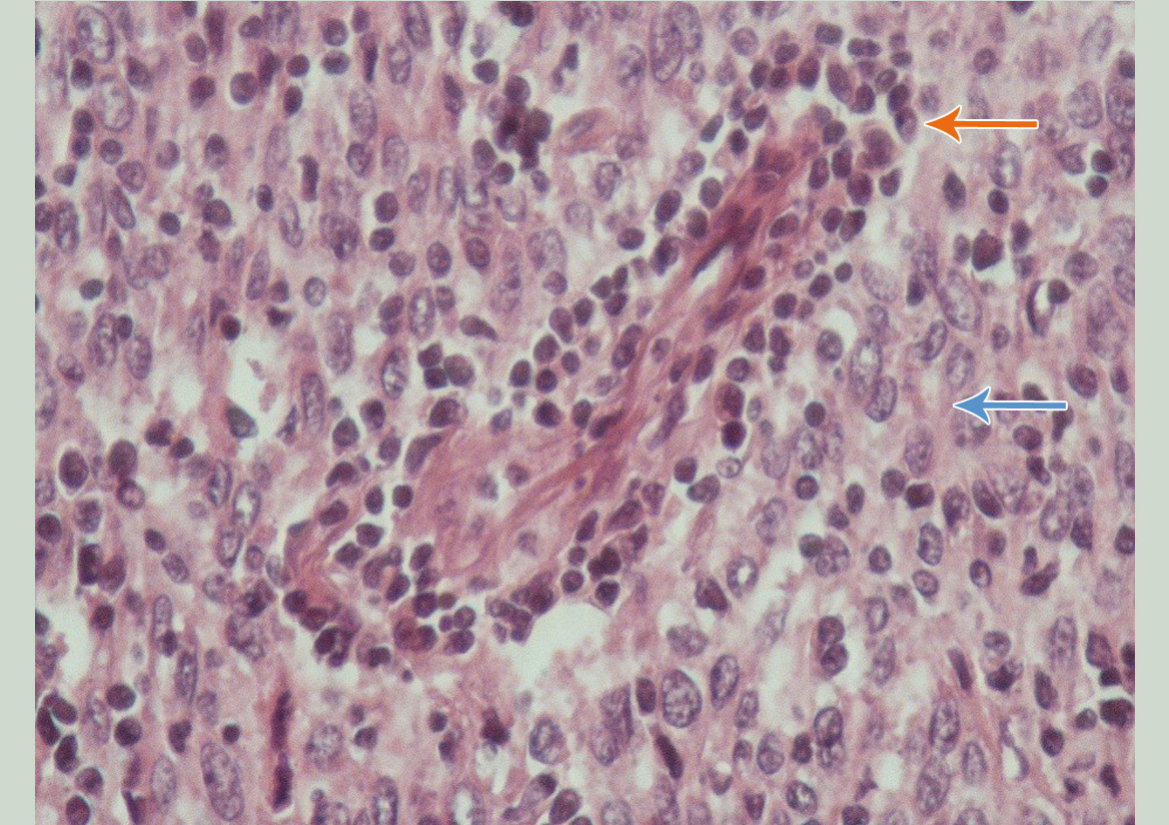
High-power view of the tumor with slightly pleomorphic neoplastic thymic epithelial cells and scattered lymphocytes (Hematoxylin & Eosin stain, × 400)

In the pathologic assessment, the microscopic examination of the prepared sections revealed a typical mixture of epithelial cells and lymphocytes with variable proportions of the 2 components throughout the lesion. The epithelial cells possessed round-to-polygonal shapes and contained vesicular nuclei with conspicuous nucleoli. The cells formed sheets and solid nests in some areas. The intermingled lymphocytic component was composed of mature lymphocytes. Small-sized vessels were present, some surrounded by a clear space containing lymphocytes and proteinaceous material (Figure 5). The immunohistochemical staining further supported the histologic diagnosis of type B3 thymoma. The epithelial component was positive for Pan-CK and focally for CD5. The labeling profile of the intermingled lymphocytes was compatible with mature T lymphocyte (positive for CD3, TdT, and CD5). The proliferative activity was 70%.

Orange arrow: lymphocytic component aggregated in the perivascular area Blue arrow: epithelial and plump cells with vesicular nuclei and conspicuous nucleoli

## Discussion

Based on the available literature, the documented prevalence of overall primary cardiac and pericardial tumors is very low (about 0.1% in autopsies)^5^ and these masses are found incidentally in most patients. 

In patients with cardiac masses, the presentation is nonspecific and is dependent on the size, site, and compressive effects of the tumor on the adjacent structures; nevertheless, the condition could prove fatal, with the first and last signs being sudden cardiac death.^5^^-^^8^ Cardiac masses are usually benign or secondary to malignant extracardiac tumors, and their definite differentiation is only possible by surgical tumor resection and tissue biopsy.^4^

Germ cell tumors (tratomas and yolk sac tumors) are the most prevalent benign tumors of the pericardium, and sarcomas are the most malignant ones. About 50% of benign pericardial tumors are tratomas, but rare cases of ectopic tumoral tissues of thyroid and thymus origins have been reported in the literature too.^9^

 Thymomas constitute a type of anterior mediastinal tumors and are asymptomatic, even in large sizes, in most patients. There have been a few cases of ectopic thymomas at unusual sites such as the neck, lung, chest wall, and pleural and pericardial space. Ectopic thymomas may originate from distributed ectopic thymic cells whose primary migration has failed. Thymomas might be symptomatic due to progressive compressive effects, invasion to the adjacent tissues, and associated immunologic disorders such as myasthenia gravis.^10^

Transthoracic echocardiographic is the most available and cost-effective noninvasive cardiac imaging, and it is used as the first choice inasmuch as it can reveal the properties of the mass such as its size, mobility, attachment site, and compressive effects.^11^

Three-dimensional echocardiography has an additive value in that it is able to assess the volumetric properties of the mass. The cropping techniques available with 3D echocardiography can help identify benign and malignant masses with regard to the homogeneity, vascularity, and calcification of the tissue.^12^

CT and cardiovascular magnetic resonance imaging, considered complementary imaging techniques to 2D and 3D echocardiography imaging, can boost diagnostic accuracy and help decision-making for the proper therapeutic approach. The individualized modality selection is essential regarding the patient’s condition and the probable diagnosis. The role of these modalities is more prominent in the detection of the invasion and other associated complications of the mass, especially as regards pericardial tumors.^13^

According to the latest literature review of ectopic thymomas in the middle mediastinum (13 reports in English), most patients were women at a mean age of 59.2 years. Only 2 patients were symptomatic, and the histologic type B3 was seen only in 1 patient.^1^ The maximum size reported thus far is 7.5 cm, while our patient’s tumor was about 13 cm in size. Its histologic type was similarly a rare form of the ectopic thymoma B3. Our review revealed rare cases of ectopic intrapericardial thymomas, and the present case adds to the existing limited literature on intrapericardial large thymomas without invading the adjacent structures. This case underscores the key role of multimodal imaging in the early diagnosis and management of cardiac tumors; be that as it may, the ultimate diagnostic strategy and the mainstay approach vis-à-vis the treatment of cardiac tumors is surgical resection and lesion biopsy.^2^^, ^^3^

Imaging studies can assist in the description of the differential diagnosis of the mass as well as its size, compressive effects, extension, and invasion characteristics, but the most accurate method for diagnosing the nature of the tumor is the pathologic assessment of the tissue. A definite diagnosis is often impossible on the basis of merely imaging findings, especially when the tumor occurs in unusual locations, and complete surgical excision without the penetration of the tumor capsule can be diagnostic and curative.^3^

## Conclusion

In light of the case presented herein, it should be borne in mind that pericardial effusion in the elderly can be a sign of malignancy and the early detection and diagnosis of associated lesions can be life-saving. Although it is not possible to determine the type of tumor in the initial evaluation, it is necessary to remember that rare different diagnoses and different cardiac imaging techniques can be helpful.

## Notes:

***To watch the following videos, please refer to the relevant URLs:***

http://jthc.tums.ac.ir/index.php/jthc/article/view/1049/890

Video 1. Severe circumferential pericardial effusion without compressive effects on the cardiac chambers in 2D transthoracic echocardiography

## Notes:

***This paper should be cited as:*** Zoroufian A, Shirzad M, Shahbazi N, Saheb Jam M, Rahnamoun Z, Shirani S, Davarpasand T. Bloody Pericardial Effusion with a Huge Pericardial Mass: A Case Report. J Teh Univ Heart Ctr 2020;15(1):35-38.

## References

[1] Yajima T, Mogi A, Shimizu K, Kosaka T, Nagashima T, Ohtaki Y, Obayashi K, Nakazawa S, Iijima M, Yoshida Y, Hirato J, Kuwano H (2018). Ectopic thymoma in the paratracheal region of the middle mediastinum: a rare case report and literature review. BMC Res Notes.

[2] Weissferdt A, Moran CA (2016). The spectrum of ectopic thymomas. Virchows Arch.

[3] Arai H, Rino Y, Fushimi K, Goda M, Yoshioka E, Okudela K, Yukawa N, Masuda M (2015). Pericardial ectopic thymoma presenting with cardiac tamponade: report of a case. Surg Today.

[4] Grebenc ML, Rosado de Christenson ML, Burke AP, Green CE, Galvin JR (2000). Primary cardiac and pericardial neoplasms: radiologic-pathologic correlation. Radiographics.

[5] Reynen K (1996). Frequency of primary tumors of the heart. Am J Cardiol.

[6] Rajiah P, Kanne JP, Kalahasti V, Schoenhagen P (2011). Computed tomography of cardiac and pericardiac masses. J Cardiovasc Comput Tomogr.

[7] Syed IS, Feng D, Harris SR, Martinez MW, Misselt AJ, Breen JF, Miller DV, Araoz PA (2008). MR imaging of cardiac masses. Magn Reson Imaging Clin N Am.

[8] Randhawa K, Ganeshan A, Hoey ET (2011). Magnetic resonance imaging of cardiac tumors: part 1, sequences, protocols, and benign tumors. Curr Probl Diagn Radiol.

[9] Burke A, Tavora F (2016). The 2015 WHO classification of tumors of the heart and pericardium. J Thorac Oncol.

[10] Lestuzzi C, Berretta M, Tomkowski W (2015). 2015 update on the diagnosis and management of neoplastic pericardial disease. Expert Rev Cardiovasc Ther.

[11] Mankad R, Herrmann J (2016). Cardiac tumors: echo assessment. Echo Res Pract.

[12] Zaragoza-Macias E, Chen MA, Gill EA (2012). Real time three-dimensional echocardiography evaluation of intracardiac masses. Echocardiography.

[13] Kassop D, Donovan MS, Cheezum MK, Nguyen BT, Gambill NB, Blankstein R, Villines TC (2014). Cardiac masses on cardiac CT: a review. Curr Cardiovasc Imaging Rep.

